# Down-regulation of the expression of CCAAT/enhancer binding protein α gene in cervical squamous cell carcinoma

**DOI:** 10.1186/1471-2407-14-417

**Published:** 2014-06-10

**Authors:** Zemin Pan, Weinan Zheng, Jinli Zhang, Rui Gao, Dongmei Li, Xiaoqing Guo, Hu Han, Feng Li, Shen Qu, Renfu Shao

**Affiliations:** 1 Department of Biochemistry and Molecular Biology, School of Medicine, Shihezi University, Xinjiang Endemic and Ethnic Disease and Education Ministry Key Laboratory, Shihezi, Xinjiang 832002, China; 2 Department of Biochemistry and Molecular Biology, Basic Medical Science of Tongji Medical College, Huazhong University of Science and Technology, Wuhan, Hubei 430030, China; 3 Genecology Research Centre, Faculty of Science, Health, Education and Engineering, University of the Sunshine Coast, Maroochydore DC, Brisbane, Queensland 4558, Australia

**Keywords:** *C/EBP*α gene, Gene expression, Cervical squamous cell carcinoma

## Abstract

**Background:**

Cervical carcinoma is the second most common cancer and is an important cause of death in women worldwide. CCAAT/enhancer binding proteins (C/EBPs) are a family of transcription factors that regulate cellular differentiation and proliferation in a variety of tissues. However, the role of *C/EBP*α gene in cervical cancer is still not clear.

**Methods:**

We investigated the expression of *C/EBP*α gene in cervical squamous cell carcinoma. *C/EBP*α mRNA level was measured by real-time quantitative RT-PCR in cervical cancer tissues and their adjacent normal tissues. C/EBPα protein level was measured by immunohistochemistry. Methylation in the promoter of *C/EBP*α gene was detected by MALDI TOF MassARRAY. We transfected HeLa cells with C/EBPα expression vector. C/EBPα expression in HeLa cells was examined and HeLa cell proliferation was measured by MTT assay and HeLa cells migration was analyzed by matrigel-coated transwell migration assays.

**Results:**

There were significant difference in C/EBPα protein expression between chronic cervicitis and cervical carcinoma (*P* < 0.001). *CEBP*α mRNA level was significantly lower in cervical cancer tissues than in normal cervical tissues (*P* < 0.01). Methylation of the promoter of *CEBP*α gene in CpG 5, CpG-14.15, CpG-19.20 were significantly higher in cervical cancer than in normal cervical tissues (*P* < 0.05, *P* < 0.01, *P* < 0.05, respectively). CEBPα pcDNA3.1 construct transfected into HeLa cells inhibited cell proliferation and decreased cell migration.

**Conclusions:**

Our results indicate that reduced *C/EBP*α gene expression may play a role in the development of cervical squamous cell carcinoma.

## Background

Cervical carcinoma is the second most common cancer and is an important cause of death in women worldwide [1]. The incidence and mortality of cervical cancer have decreased gradually in the past decades globally. In China, however, the incidence of cervical cancer remains high, particularly in young women [2].

CCAAT/enhancer binding proteins (C/EBPs) are a family of transcription factors with six members, α to ζ; C/EBPs regulate cellular differentiation in a variety of tissues [3]. Each C/EBP consists of an activation domain, a DNA-binding domain, and a leucine-rich dimerization domain. C/EBPα protein interacts with several cell-cycle regulatory proteins; such interaction can inhibit cell proliferation. For instance, C/EBPα interacts with retinoblastoma (Rb) family proteins and inhibits cell growth [4]. Studies showed that C/EBPα can form a complex with cyclin-dependent kinase 2 (cdk2) and cyclin-dependent kinase 4 (cdk4) proteins and block cyclin-cdk interactions and cell cycle progression [5]. Aberrant expression of *C/EBPα* in Trib1-deficient bone marrow cells is responsible for the defects in macrophage differentiation [6]. In addition, it was suggested that high levels of *C/EBPα* accelerate both the switching process and the cell growth arrest [7]. *C/EBPα* gene was down-regulated in many tumors such as skin carcinomas, breast cancer and lung cancer [8]. *C/EBPα* gene as a lung tumor suppressor was demonstrated: loss of *C/EBPα* expression through p38α inactivation led to tumor promotion and progression [9].

In our early work, we used suppression subtractive hybridization method and found *C/EBP*α gene expression changed in cervical carcinoma tissues [10]. In addition, Ko found that *C/EBPδ* gene expression level decreased in cervical cancer [11]. We show here that *C/EBP*α gene is also down-regulated in cervical squamous cell carcinoma (CSCC).

## Methods

### Sample collection

Clinical data and cervical squamous cell carcinoma samples were collected from patients at the First Affiliated Hospital and the Third Affiliated Hospital of the Medical College of Shihezi University, Xinjiang, China between January 2008 and April 2012. Cervical cancer samples were taken for every consecutive patient with different grades of pathology. None of these patients received chemotherapy or radiotherapy before the cervical tissue samples were obtained. All histological diagnoses were confirmed by experienced pathologists in the hospitals. Written informed consent was obtained from each patient; approval was obtained from the Ethics Committee of the Medical College of Shihezi University, China. Cervical squamous cell carcinoma tissues and adjacent normal cervical tissues were collected together from each patient. Tissue samples were snap-frozen immediately after removal and were stored at -80°C.

### Immunohistochemistry staining

Representative formalin fixed paraffin-embedded tissue blocks were selected. Five μm sections were cut, deparaffinised and rehydrated through graded alcohols. Antigen retrieval was performed by heating the slides in citrate buffer at 98°C for 30 min in a water bath. Endogenous peroxidase was quenched for 10 min with peroxidase blocking reagent (Dako Corporation). Primary antibodies, anti-C/EBPα (1:200; sc-61, Santa Cruz Biotechnology) and anti-Ki-67 (1:100; BD Biosciences Pharmingen) were incubated for 60 min at room temperature. Antibody staining was visualized using the ChemMate Envision detection system (Dako Cytomation). Sections were counterstained. The counterstaining was performed with Meyer’s hematoxylin solution. Negative controls were run simultaneously with preimmune immunoglobulin. Rabbit polyclonal antibody (Ab-1) used was diluted 10- and 50-fold. The specificity of the immunohistochemical reactions was assessed by performing the assays in the presence of an excess of relevant versus irrelevant peptides. The peptide used for immunization completely suppressed staining whereas an irrelevant peptide at the same concentration had no effect. The C/EBPα protein and Ki-67 protein IHC signal was scored on the following scale taking into account both the proportion of cells stained and the intensity of staining in those cells as follows: score 0, no cells stained; score 1, weak or absent nuclear staining and <5% of cells containing C/EBPα and Ki-67 localized to the nuclei of cells; score 2, nuclear staining and between 5% and 25% of the cells containing C/EBPα and Ki-67 localized prominently to the nuclei of cells; score 3, nuclear staining and between 26% and 50% of the cells containing C/EBPα and Ki-67 localized prominently to the nuclei of cells; score 4, nuclear staining and more than 50% of the cells containing C/EBPα and Ki-67 localized prominently to the nuclei of cells. Two observers quantified independently.

### Quantitative reverse transcription-PCR (qRT-PCR)

Total RNA was isolated from cervical squamous cell carcinoma tissues and their adjacent normal tissues using the RNA Extraction Kit (Invitrogen). cDNA were synthesized with Invitrogen's SuperScript One-Step RT-PCR Kit; each reaction contained 2 μg total RNA, 2 μl Oligo(dT) (500 μg/ml), and 7.5 μl DEPC water. Reactions were heated for denaturation at 65°C for 5 min, then quenched on ice for 5 min. The following reagents were then added to each reaction: 4 μl 5× 1st Buffer, 2 ul 0.1 M DTT, 1 μl dNTPs (10 mM each), 0.5 μl RNase Inhibitor (40 U/μl), 1 μl M-MLV (200 U/μl); the total volume of each reaction was 20 μl. The reactions were kept at 25°C for 10 min, 37°C 1 h, and then 70°C for 10 min to terminate the reaction. *C/EBPα* mRNA level was determined by real-time RT-PCR using a Light Cycler 480 (Roche Diagnostics) with the forward primer, 5′-AACACGAAGCACGATCAGTCC-3′, and the reverse primer, 5′-CTCATTTTGGCAAGTATCCGA-3′. The amplicons were 211 bp in size. To normalize the amount of cDNA in each sample, the housekeeping gene aldehyde-3-phosphate dehydrogenase (GAPDH) was quantified on the control of experiment with specific primers (forward: 5′-TGTTGCCATCAATGACCCCTT-3′; reverse: 5′-CTCCACGACGTACTCAGCG-3′); the amplicons were 202 bp. Each reaction contained cDNA 500 ng, 2× PCR buffer for EvaGreen 10 μl, 20× EvaGreen 0.6 μl, forward primer and reverse primer were 0.6 μl (10 uM/) respectively, Cap Taq polymerase 0.3 μl (5 U/μl); add DEPC water to 20 μl. Reaction conditions were: initial denaturation for 5 min at 95°C; then 40 cycles of denaturation for 15 sec at 95°C, primer annealing for 15 sec at 55°C, extension for 20 sec at 72°C, and UPL fluorescence measurement for 3 sec at 76°C.

### Gene methylation analysis by matrix assisted laser desorption ionization time of flight MassARRAY (MALDI-TOF MassARRAY)

Gene methylation was analyzed using MALDI-TOF MassARRAY method [12]. DNA from cervical tissue samples was extracted using QIAamp DNA Mini Kit (QIAGEN). Bisulfite treatment was performed using EZ DNA methylation kit (Sequenom, USA). PCR was performed in a total volume of 5 μl containing 0.5 U Hot Star Taq polymerase (Qiagen), 10 pmol of forward and reverse primers 0.5 μl, 10× PCR buffer (Mg^2+^ free) 0.5 μl, ddH_2_O 0.5 μl, MgCl_2_ 0.4 μl, 25mM dNTP 0.1 μl and 5 ng template. Cycling was performed using a Mastercycler (Eppendorf) under the following conditions: 15 min at 94°C; 45 cycles at 94°C for 20 sec, annealing at 62°C for 30 sec and extension 72°C for 1 min; and finally, extension at 72°C for 3 min. Shrimp alkaline phosphotase (SAP) mixture (2 μl) was then added to each reaction. The reactions were vortexed briefly, centrifuged at 3,000 rpm for 5 min, and incubated at 37°C for 20 min, and 85°C for 5 min.

RNA transcription was performed in a volume of 5 μl containing RNase Free-ddH_2_O (3.15 μl), 5× T cleavage & Polymerase buffer (0.89 μl), T7 RNA & DNA polymerase (0.44 μl), T cleavage mix (0.24 μl), DTT (100 mM, 0.22 μl), and RNAase A (0.06 μl). After incubation at 37°C for 3 hr, 6 mg Clean Resin (Sequenom) was added to desalt RNA. MassARRAY MS (Bruker-Sequenom USA) was used to analyze the data.

### Cell culture and transfection

cDNA of *C/EBPα* gene was cloned into pcDNA3.1(-) vector and confirmed by sequencing after cloning into the pcDNA3.1(-) expression vector. HeLa cells were initially obtained from ATCC (American Type Culture Collection, Manassas, VA) and maintained in our lab. Non-transfected HeLa cells and those transfected with pcDNA3.1(-) were used as controls. HeLa cells were plated at 1–2 × 10^5^ cells per well in a six-well cell culture plate 12–24 hr before the transfection. Two μg of C/EBPα constructs and control plasmids pcDNA3.1 were mixed with 6 μl of Lipofect AMINE 2000 (Invitrogen). The mixture was incubated at room temperature for 20 min. After washing the cells with 1× PBS, the DNA/ Lipofect AMINE 2000 mixtures were transferred to HeLa cells. Plasmids pcDNA3.1 were also transferred into separate HeLa cells as controls. Transfected HeLa cells were then incubated at 37°C in the 5% CO_2_ for 24 hr. The effect of C/EBPα overexpression was determined by measuring immunofluorescence luciferase activity using an assay system according to the manufacturer’s protocol (Promega). Each experiment was repeated with multiple batches of cells.

### Cell survival rate assay using MTT

The survival rate of HeLa cells transfected by C/EBPα pcDNA3.1 construct and pcDNA3.1, and non-transfected HeLa cells, was determined using the 3-(3,4-dimethyl-thiazol-2-yl)-2,5-diphenyltetrazolium bromide (MTT) assay. The HeLa cells transfected with C/EBPα pcDNA3.1 constructs, transfected cells with pcDNA3.1, and non-transfected HeLa Cells were seeded in 96-well plates (Falcon; Becton Dickinson Labware) at 0.6 × 10^4^ cells per well in DMEM containing 10% FBS. The cells were incubated for 24 hr at 37°C; the number of cells was quantified with the MTT cell growth assay kit according to the manufacturer’s instruction. Briefly, 20 μl of MTT solution was added to each well, incubated for 4 hr, and then scanned at 570 nm by microplate reader MTP-300 (Corona); the quantification was repeated 10 times. The statistics were obtained from ten individual experiments by analysis of variance.

### Cell migration assays

Cells were serum-starved overnight. The top chambers of 6.5 mm Corning Costar transwells (Corning, NY, New York, USA) were loaded with 0.2 ml of cells (5 × 10^5^ cells/ml) in serum-free media. DMEM media containing 20% FBS 0.6 ml was added to the bottom wells and the cells were then incubated at 37°C overnight. Cells on the top layer were removed and the images of the cells at the bottom of the membrane were captured using a canon camera microscope (×200). The statistics were obtained from five individual experiments by analysis of variance.

### Statistics

Statistical analysis was performed using SPSS 17.0 software. C/EBPα protein levels were compared between cervical carcinoma and chronic cervicitis using non-parametric test. *C/EBP*α mRNA level in cervical carcinoma tissues and the adjacent normal tissues was analyzed by Student ‘t’ test. The promoter of *C/EBP*α gene methylation by MALDI TOF MassARRAY method was analyzed with Wilcoxon Rank Sum Test.

## Results

### C/EBPα protein level in cervical carcinoma tissues and chronic cervicitis tissues

**Figure 1:**
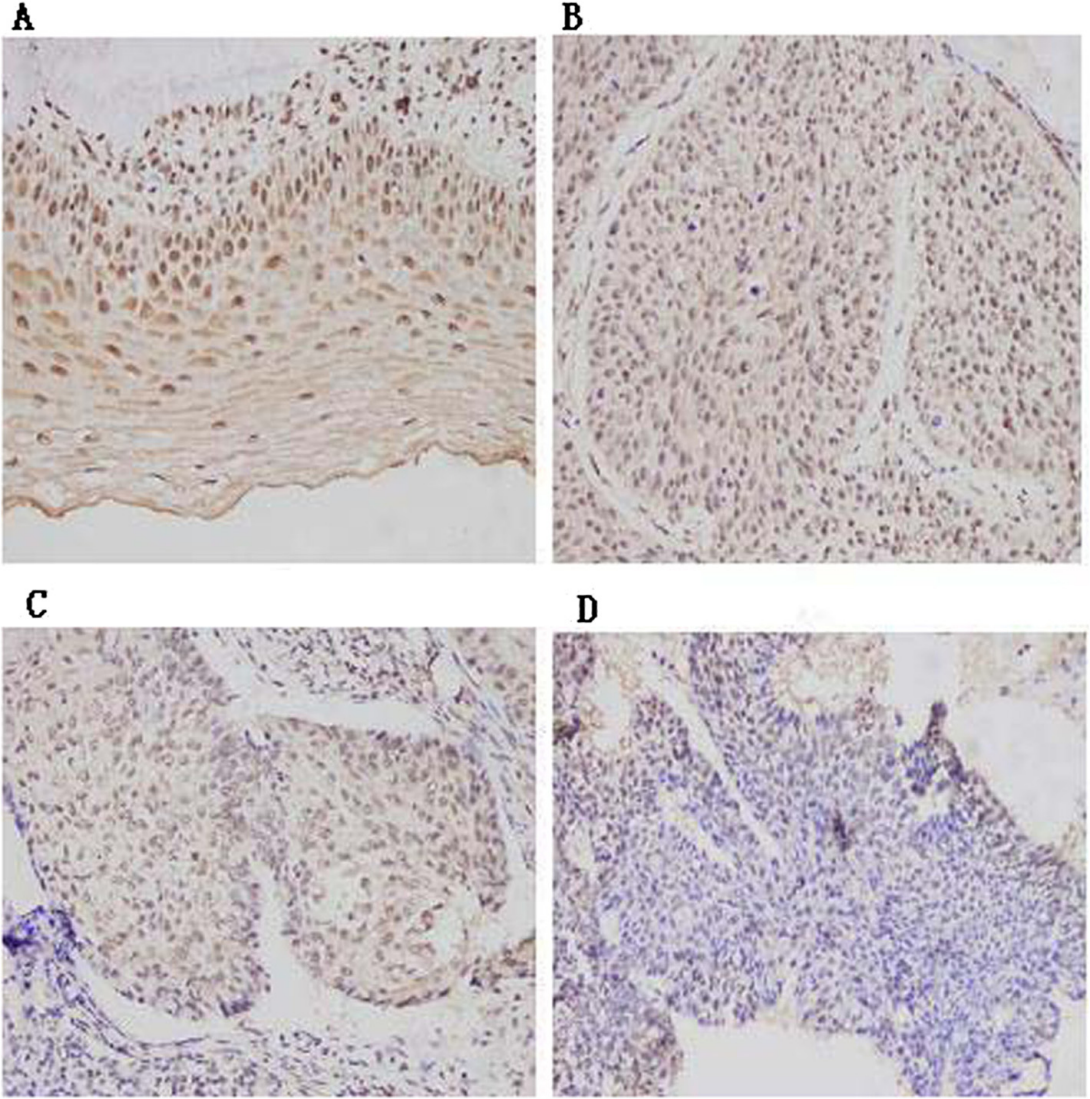
**Expression level of C/EBPα protein in chronic cervicitis tissues and cervical carcinoma measured by immunohistochemistry (200× Magnification). (A)** chronic cervicitis **(B)** well-differentiated cancer **(C)** moderately differentiated cancer **(D)** poorly differentiated cancer.

**Figure 2:**
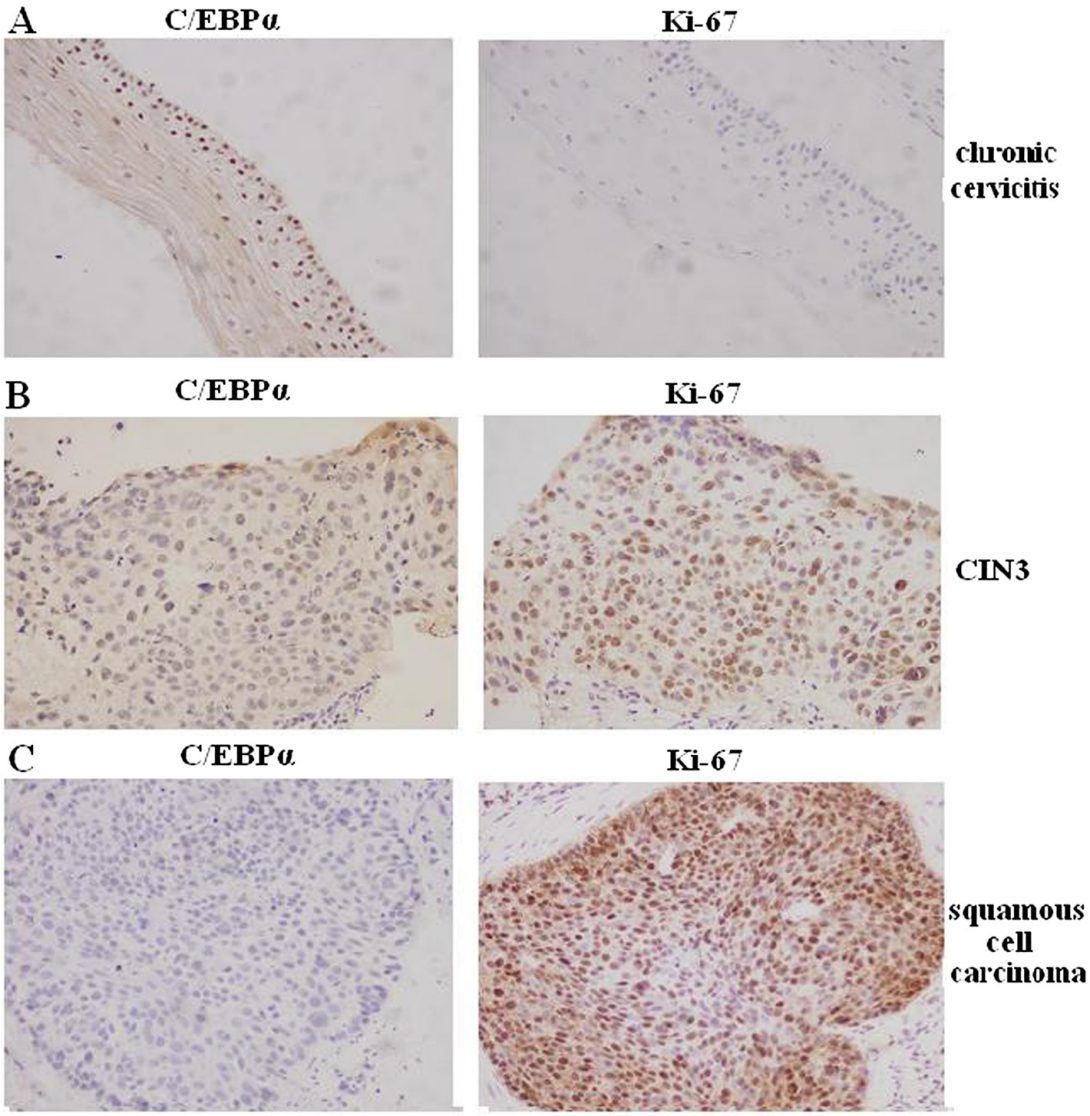
**C/EBPα staining coincides with cell proliferation related protein Ki-67. (A)** Two serial sections of a chronic cervicitis sample stained with C/EBPα, Ki-67 antibodies as indicated (200× Magnification). **(B)** Two serial sections of the same sample showing CIN3 are stained with C/EBPα, Ki-67 antibodies as indicated (200× Magnification). **(C)** Two serial sections of the same sample showing squamous cell carcinoma are stained with C/EBPα, Ki-67 antibodies as indicated (200× Magnification).

C/EBPα protein level was strongly positive (score 4) in the nuclei of chronic cervicitis cells. There were 70 strong staining (score 4), 19 medium staining (score 3), 20 weak staining (score 2) and 16 negative staining (score 0–1) in chronic cervicitis tissues. There were, however, 44 strong staining (score 4), 59 medium staining (score 3), 34 weak staining (score 2) and 22 negative staining (score 0–1) in cervical carcinoma. C/EBPα protein level was the highest in well-differentiated cervical carcinoma, followed by moderately differentiated cervical cancer, and was the lowest in poorly differentiated cervical cancer (Figure 1). Thus, the difference between chronic cervicitis and cervical carcinoma in C/EBPα protein level was significant (*P* < 0.001) (Table 1). C/EBPα protein level in the well-differentiated cervical carcinoma was significantly higher than that in the moderately differentiated cervical carcinoma. Further, C/EBPα protein level in moderately differentiated cervical carcinoma was significantly higher than that in poorly differentiated cervical carcinoma (*P* < 0.001) (Table 2). Immunohistochemistry staining of serial sections of tissues showed that expression of C/EBPα protein was increased whereas expression of Ki-67 protein expression was reduced in chronic cervicitis. However, in CIN3 (carcinoma in situ) and squamous cell carcinoma, C/EBPα protein expression was decreased whereas Ki-67 protein expression was increased (Figure 2). Therefore, it is possible that C/EBPα protein could regulate cervical cell proliferation.

**Table 1:** Expression of C/EBPα protein in the chronic cervicitis tissues and cervical carcinoma tissues

**Tissues**	**Number**	**Score 4**	**Score 3**	**Score 2**	**Score 0-1**	
Chronic cervicitis tissues	125	70	19	20	16	*P* < 0.001
Cervical squamous cell carcinoma tissues	159	44	59	34	22	

**Table 2:** Expression level of C/EBPα protein in cervical squamous carcinoma of different grades of pathology

**Tissues**	**Number**	**C/EBPα protein expression**				
		**Score 4**	**Score 3**	**Score 2**	**Score 0-1**	
Well differentiated carcinoma	38	13	9	10	6	*P* < 0.001
Moderately differentiated carcinoma	99	31	49	10	9	
Poorly differentiated carcinoma	22	0	1	14	7	

### *C/EBP*α mRNA level in cervical carcinoma and normal cervical tissues

**Figure 3:**
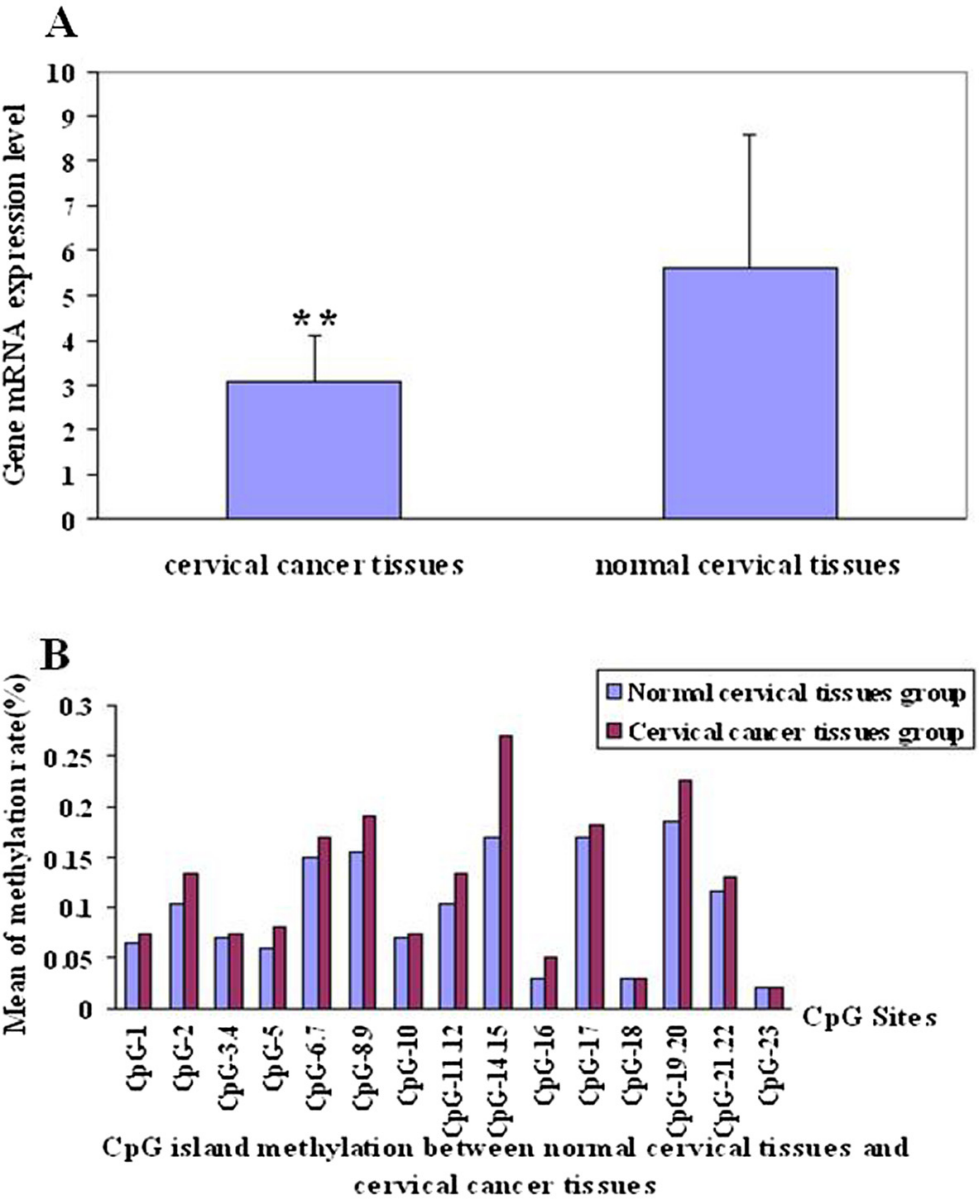
**Expression level of C/EBPα mRNA in cervical tissues. (A)** C/EBPα mRNA expression was significantly reduced in cervical cancer tissues than in normal cervical tissues, (*P* < 0.01) and marked by an asterisk. **(B)** The promoter of C/EBPα gene methylation rate in CpG 5, CpG-14.15, CpG-19.20 were significantly higher than in normal cervical tissues (*P* < 0.05, *P* < 0.01, *P* < 0.05, respectively).

Fifteen cervical carcinoma tissues and their corresponding normal cervical tissues were examined for *CEBP*α gene expression by real time quantitative RT-PCR. The average *CEBP*α mRNA level was 3.07 ± 1.04 in cervical cancer tissues and 5.63 ± 2.98 in their corresponding normal cervical tissues (Figure 3A). The difference between cervical tissues and their corresponding normal cervical tissues was significant (*t* = -3.150, *P* < 0.01). Thus, the expression of *CEBP*α gene was reduced significantly in cervical carcinoma tissues.

### Methylation in the promoter of *C/EBP*α gene analysis

Forty-seven cervical carcinoma tissues and 25 normal tissues were analyzed for methylation in the promoter of *C/EBPα* gene by MALDI TOF MassARRAY. We found that the rate of *C/EBPα* gene methylation in CpG 5, CpG-14.15, CpG-19.20 were significantly higher in cervical tissues than in normal cervical tissues (*P* < 0.05, *P* < 0.01, *P* < 0.05, respectively; Figure 3B). Methylation in the promoter of *C/EBPα* gene, thus, may have caused the reduction in the expression of this gene.

### *C/EBPα* gene construct transfected into HeLa cells inhibit cell growth and decrease cell migration

**Figure 4:**
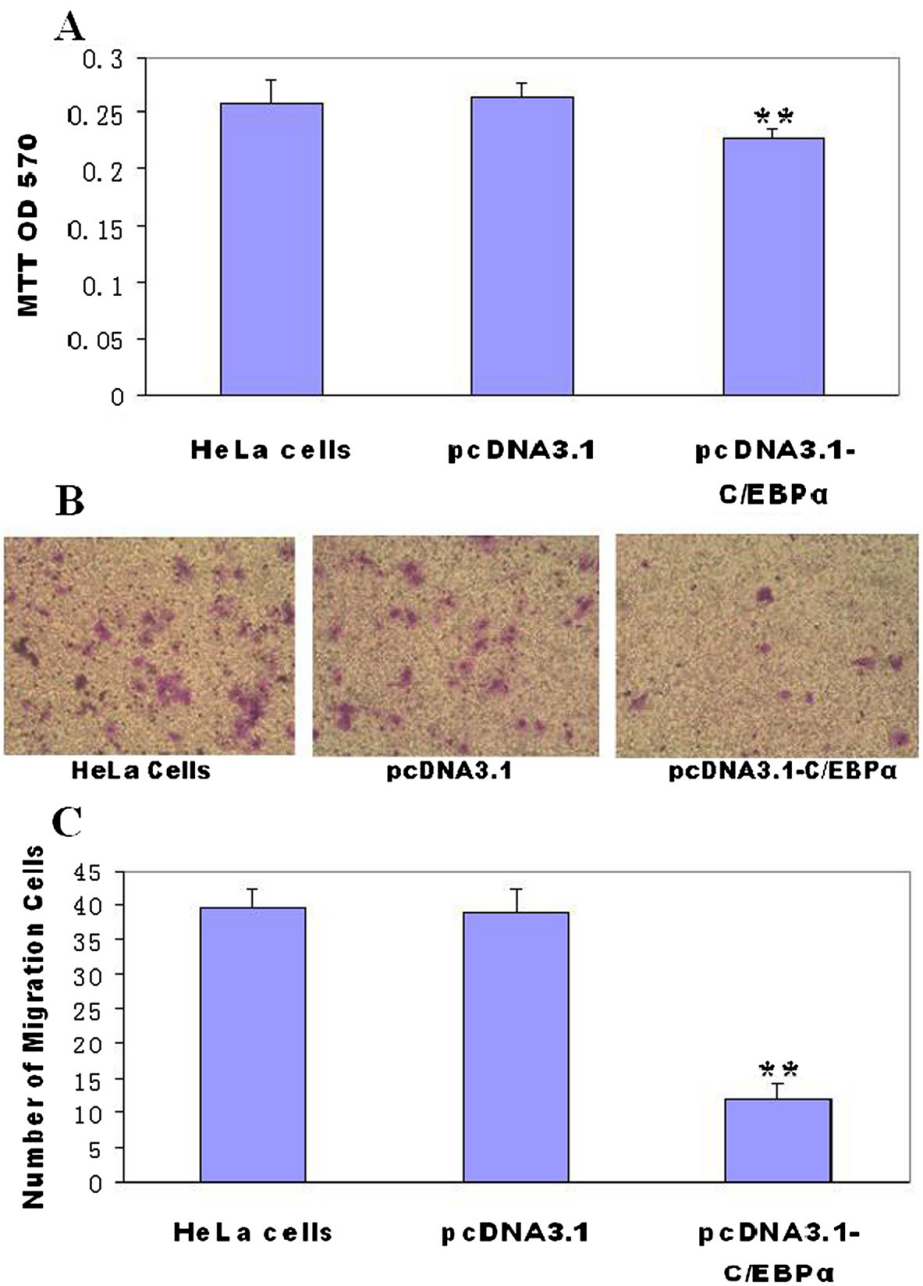
**Inhibited growth and decreased migration of HeLa cells transfected with C/EBPα construct. (A)** MTT Assay: equal numbers of HeLa cells transfected with either empty vector (pcDNA3.1) and C/EBPα expression vector (pcDNA3.1- C/EBPα), and non-transfected cells (HeLa cells), were grown in the serum-free DMEM medium. After 24 hours, cell proliferation was determined by MTT assays. Cell numbers between pcDNA3.1-C/EBPα transfected groups and between pcDNA3.1 transfected and non-transfected groups were significantly different with mean ± SD (n = 10) (*P* < 0.001). **(B)** The photographs show a representative field from each of the cell lines that have migrated into the chamber. pcDNA3.1-C/EBPα group cell migration is reduced than pcDNA3.1 vector and non transfected HeLa cells. **(C)** In the lower panel, The number of migration of HeLa cells is calculated among three groups. C/EBPα reduced migration is statistically significant with mean ± SD (n = 5) (*P* < 0.001), and marked by an asterisk.

The proliferation of HeLa cells transfected by C/EBPα pcDNA3.1 construct was inhibited significantly compared to those transfected by pcDNA3.1 plasmid and non-transfected HeLa cells using MTT assay (*P* < 0.001) (Figure 4A). This study indicates that *C/EBPα* gene may inhibit HeLa cell proliferation. To assess whether overexpression of C/EBPα is sufficient to reduce cell migration, we measured the migration of HeLa cells using transwell migration assays. The number of HeLa cells migrated in C/EBPα pcDNA3.1 construct transfected group is significantly less than that in the two control groups, i.e. pcDNA3.1 plasmid transfected group (without C/EBPα gene) and the HeLa cells not subject to transfection (Figure 4B). Thus, the expression of C/EBPα protein in HeLa appears to inhibit cell migration. The numbers of migration cells are significantly different between C/EBPα pcDNA3.1 construct transfected group and the two control groups with mean ± SD (n = 5) (*P* < 0.001) (Figure 4C).

## Discussion

Proteins in the C/EBP family are fundamental to the control of differentiation and proliferation of many adult tissues. *C/EBPα* gene is involved in mitotic growth arrest and differentiation of numerous cell types. Down-regulation of *C/EBPα* in keratinocytes can be permissive for cell proliferation and may block squamous differentiation. Reduction of *C/EBPα* gene expression occurred in hepatocellular carcinoma, skin carcinoma and lung cancer, in particular in lung adenocarcinomas, which had the most significant and frequent reduction in *C/EBPα* gene expression [4]. *C/EBPα* is aberrantly silenced in pancreatic cancer cells and restored *C/EBPα* gene expression markedly suppressed proliferation of pancreatic cancer cells [13]. In gastric carcinoma, loss of C/EBPα is associated with the switch from cellular differentiation to cellular proliferation, presumably as a result of the activation of Ras/MAPK pathway [14]. In addition, methylation in the promoter of *C/EBPα* gene occurred at a rate of 24% in dedifferentiated liposarcoma (DLPS). While treatment with demethylating agents could restore *C/EBPα* expression in DLPS cells, it was anti-proliferative and pro-apoptotic in vitro and reduced tumor growth in vivo [15]. *C/EBPα* gene is located in 19q13, gene loss at this site of chromsome was associated with local recurrence of dedifferentiated liposarcoma (DDLS) [16].

Disease-specific survival was shorter for patients with 19q13 loss than for patients with diploid 19q13 [16]. Common copy-number losses were associated with transcriptional down-regulation of *C/EBPα* gene [16]. *C/EBPα* can interact with some genes and affect cell activities [17]. Restoration of *C/EBPα* in Yin-Yang-1 (YY1) gene expressing hepatocellular carcinoma (HCC) cells induced cellular differentiation and growth inhibition, while knockdown of *C/EBPα* expression in non-tumor liver cells promoted cell growth. Therefore, it indicted that YY1 gene could promote hepatocellular carcinogenesis and inhibit cellular differentiation through the down-regulation of *C/EBPα* expression [17]. There is evidence that *C/EBPα* exerts its effects, in part, by regulating specific micro RNAs, such as miR-223 [18]. miR-511 and miR-1297 act as tumor suppressor genes, which could suppress lung adenocarcinoma A549 cell line proliferation in vitro and in vivo by suppressing tribbles homolog 2 *(TRIB2)* gene and further increasing *C/EBPα* gene expression [19]. It showed that NAD(P)H:quinone oxidoreductase 1 (NQO1) gene control of *C/EBPα* against 20S proteasome degradation contributed to the up-regulation of p63 expression and protection for thinning of the epithelium and chemical-induced skin cancer [20].

In the mouse model of AML, C-terminal C/EBPα mutations increase the proliferation of long-term hematopoietic stem cells (LT-HSC) whereas N-terminal C/EBPα mutations allow formation of leukemia initiating cells [21]. The evidence available indicates that impaired C/EBPα function contributes directly to the development of AML; thus restoring C/EBPα function represents a promising target for novel therapeutic strategies in AML [22]. The consequential up-regulation of C/EBPα and IGFBP-5 by curcumin is crucial to the suppression of oral carcinogenesis [23]. In acute promyelocytic leukemia cancer-initiating cells, *C/EBPα* expression is down-regulated, possibly through a methylation-dependent mechanism, indicating that *C/EBPα* deregulation may contribute to the transformation of these cells [24]. The ectopic expression of *C/EBPα* can induce the monocytic differentiation of myelomonocytic leukemic cells through the down-regulation of Myc gene [25].

The expression of *C/EBPα* gene in cervical cancer is poorly understood. We found that C/EBPα protein expression was significantly different between chronic cervicitis and cervical squamous cell carcinoma. Moreover, the expression level of C/EBPα protein in the well-differentiated cervical carcinoma was significantly higher than that in the moderately differentiated cervical carcinoma. Furthermore, the expression level of C/EBPα protein in the moderately differentiated cervical carcinoma was higher than that in the poorly differentiated cervical carcinoma. Clearly, C/EBPα protein expression level was related to the grade of pathology. Our results indicate that we may be able to interfere the carcinogenesis of cervical cancer by regulating the expression of *C/EBPα* gene. Thus, C/EBPα protein could potentially be a target for cervical cancer treatment and changes in the expression of C/EBPα protein could be of significance to the early diagnosis of cervical cancer.In addition, C/EBPα protein increased expression whereas Ki-67 protein decreased expression in chronic cervicitis tissues. However, C/EBPα protein decreased expression whereas Ki-67 protein increased expression in cancer tissues (Figure 2). Thus, C/EBPα may affect cervical cell proliferation. These results are similar to that obtained by MTT assay of HeLa cells (Figure 4C).

We analyzed the *C/EBP*α mRNA expression in cervical squamous cell carcinoma and their corresponding normal tissues. The *C/EBP*α mRNA expression level was significantly higher in cervical squamous cell carcinoma tissues than in their corresponding normal cervical tissues. Our results indicated that *C/EBPα* mRNA expression level was decreased in invasive cervical cancer. In addition, methylation in the promoter of *C/EBPα* gene in cervical cancer tissues was significantly higher than in normal cervical tissues. It is possible that the methylation in the promoter of *C/EBPα* gene leads to decreased expression of this gene.

The full length mRNA of *C/EBPα* gene was cloned in pcDNA3.1 eukaryotic expression vector. The *C/EBPα* gene construct and pcDNA3.1 vector were transfected into HeLa cells. HeLa cells transfected by *C/EBPα* gene construct were significantly lower in growth than the HeLa cells transfected by pcDNA3.1 plasmid and the non-transfected HeLa cells by MTT assay. In addition, after being transfected by *C/EBPα* gene construct, the migration of HeLa cells was significantly reduced than those transfected by pcDNA3.1 plasmid and the non-transfected HeLa cells by matrigel-coated transwell migration assays. These results indicate that *C/EBPα* gene may inhibit the growth of HeLa cells and decrease the invasion of HeLa cells. Therefore, *C/EBPα* gene may act as a tumor suppressor gene for cervical carcinoma.

## Conclusions

In summary, the expression of *C/EBP*α gene and C/EBPα protein was down-regulated in cervical carcinoma tissues, possibly caused by methylation in the promoter region of this gene. Reduced expression of *C/EBP*α gene and C/EBPα protein appears to be associated with cervical tumorigenesis. By gene transfection methods, we showed that *C/EBPα* gene could inhibit the growth of HeLa cells and reduce the invasion of HeLa cells. Therefore, *C/EBPα* gene may be a tumor suppressor gene in HeLa cells and plays an important role in cervical carcinogenesis.

## Competing interests

The authors declare that they have no competing interests.

## Authors’ contributions

ZP carried out the molecular genetic studies, participated in the gene analysis and wrote the manuscript. WZ carried out the immunohistochemistry Staining. JZ and DL carried out the qRT-PCR. RG and XG carried out gene methylation analysis. HH carried out gene transfection. FL participated in the design of the study and performed the statistical analysis. SQ and RS participated in experimental design and wrote the manuscript. All authors read and approved the final manuscript.

## Pre-publication history

The pre-publication history for this paper can be accessed here:

http://www.biomedcentral.com/1471-2407/14/417/prepub
